# Effects of Different Formulations of Glyphosate on Rumen Microbial Metabolism and Bacterial Community Composition in the Rumen Simulation Technique System

**DOI:** 10.3389/fmicb.2022.873101

**Published:** 2022-04-29

**Authors:** Melanie Brede, Sven-Bastiaan Haange, Susanne Riede, Beatrice Engelmann, Nico Jehmlich, Ulrike Rolle-Kampzczyk, Karl Rohn, Dirk von Soosten, Martin von Bergen, Gerhard Breves

**Affiliations:** ^1^Institute for Physiology and Cell Biology, University of Veterinary Medicine, Hannover, Germany; ^2^Department of Molecular Systems Biology, Helmholtz Centre for Environmental Research (UFZ), Leipzig, Germany; ^3^Institute for Biometry, Epidemiology and Information Processing, University of Veterinary Medicine, Hannover, Germany; ^4^Institute of Animal Nutrition, Friedrich-Loeffler-Institut (FLI), Federal Research Institute for Animal Health, Brunswick, Germany; ^5^German Centre for Integrative Biodiversity Research (iDiv) Halle-Jena-Leipzig, Leipzig, Germany; ^6^Faculty of Life Sciences, Institute of Biochemistry, University of Leipzig, Leipzig, Germany

**Keywords:** rumen, glyphosate, fermentation, metaproteome, bacterial community, 16S rRNA gene sequencing

## Abstract

The use of the herbicide glyphosate and its formulations on protein-rich feedstuff for cattle leads to a considerable intake of glyphosate into the rumen of the animals, where glyphosate may potentially impair the 5-enolpyruvylshikimate-3-phosphate pathway of the commensal microbiota, which could cause dysbiosis or proliferation of pathogenic microorganisms. Here, we evaluated the effects of pure glyphosate and the formulations Durano TF and Roundup^®^ LB plus in different concentrations on the fermentation pattern, community composition and metabolic activity of the rumen microbiota using the Rumen Simulation Technique (RUSITEC). Application of the compounds in three concentrations (0.1 mg/l, 1.0 mg/l or 10 mg/l, *n* = 4 each) for 9 days did not affect fermentation parameters such as pH, redox potential, NH_3_-N concentration and production of short-chain fatty acids compared to a control group. Microbial protein synthesis and the degradation of different feed fractions did not vary among the treatments. None of the used compounds or concentrations did affect the microbial diversity or abundance of microbial taxa. Metaproteomics revealed that the present metabolic pathways including the shikimate pathway were not affected by addition of glyphosate, Durano TF or Roundup^®^ LB plus. In conclusion, neither pure glyphosate, nor its formulations Durano TF and Roundup^®^ LB plus did affect the bacterial communities of the rumen.

## Introduction

Glyphosate is an herbicide acting by inhibition of the 5-enolpyruvylshikimate-3-phosphate pathway (Amrhein et al., 1980a; Boocock and Coggins, 1983). Glyphosate-containing herbicides are among the most-used herbicides worldwide. Glyphosate use in the United States has increased 300-fold in 30 years since its market introduction. Due to the development of glyphosate-resistant crops (Benbrook, 2016), the use of this herbicide is especially popular in soybean and maize production. In Europe, the application of glyphosate is being intensely discussed in regard to potential detrimental environmental or health effects, though it still accounts for one-third of the herbicide volume in 2017 (Antier et al., 2020). The mode of action of Glyphosate is that it directly inhibits 5-enolpyruvylshikimate-3-phosphate synthase and therefore hinders aromatic amino acid synthesis (Amrhein et al., 1980b). Although the 5-enolpyruvylshikimate-3-phosphate pathway is not present in human and animal cells, the microbial community of the gastrointestinal tract of animals and humans also contains glyphosate-sensitive members (Motta et al., 2018; Leino et al., 2021; Mesnage et al., 2021). In honeybees, for example glyphosate decreased the abundance of *Snodgrasella alvi* favouring infection with *Serratia marescens* but not with *Nosema ceranae* (Motta et al., 2018; Blot et al., 2019).

Ruminants livestock are fed protein-rich feed components such as rapes and soybeans which are highly exposed to glyphosate (Benbrook, 2016; Antier et al., 2020). In ruminants, the microbial community of the forestomach is vital in the degradation of plant-derived carbohydrates to short-chain fatty acids (SCFA), a major energy source for the host, as well as providing the host with microbially synthesised protein and water soluble vitamins. Glyphosate could potentially affect and disrupt the microbial community in the forestomach, and therefore might lead to an adverse health outcome for the host. Earlier, several reports on an association between glyphosate and so-called ‘visceral botulism’ in cows (Krüger et al., 2013; Ackermann et al., 2015) have been published.

*In vivo*, von Soosten et al. (2016) measured a glyphosate intake between 0.08 mg/d and 6.67 mg/d in dairy cows under conventional feeding conditions, of which 61% were excreted by faeces, but 6–36% disappeared during rumen passage indicating a potential interaction or degradation by rumen microorganisms. In sheep, Hüther et al. (2005) did not observe effects of feeding 0.77 g/kg of dry matter glyphosate on rumen fermentation parameters, such as pH or SCFA concentration, or *in sacco* degradation of feedstuff. Using the Rumen simulation technique (RUSITEC) as *in vitro* model for rumen fermentation, Riede et al. (2016) reported a decrease in NH_3_-N concentrations and a trend towards increasing acetate production; however, glyphosate application (3.31 mg/l) did not affect the composition of the *Clostridia* population. High glyphosate levels (10 mg/l) did also not affect establishment of *Escherichia coli* or *Salmonella* Ser. *Typhimurium* in the RUSITEC system (Bote et al., 2019). Schnabel et al. (2017) did not observe adverse effects of a glyphosate-contaminated diet (intake up to 84.5 mg/d) on performance or energy balance of dairy cows, neither did this affect leucocytes or erythrocytes (Schnabel et al., 2020) or the rumen microbiome (Billenkamp et al., 2021).

Commercial herbicides contain a mixture of glyphosate and adjuvants, e.g., polyethoxylene amine (POEA), which are assumed to enhance toxicity of glyphosate or exhibit additional effects themselves (Defarge et al., 2018; Mesnage et al., 2019). Different formulations vary regarding their effects on the gut community and their metabolome profile in rats (Mesnage et al., 2021). To our knowledge, no data comparative on the effects of different glyphosate formulations on the rumen microbiome are available and this might account for differences in the results of previous studies. In this study, we compare the effects of pure glyphosate and two widely used commercial formulations on the rumen biochemical functions as well as rumen microbiome taxonomic composition and functionality at concentrations comparable to and above the observed *in vivo* levels reported by von Soosten et al. (2016).

## Materials and Methods

### Animals

Rumen contents were obtained from four ruminal fistulated German Holstein cattle housed at the Institute for Animal Nutrition, Federal Research Institute for Animal Nutrition, Brunswick, Germany. Fistulation of the animals was approved by the Lower Saxony State Office for Costumer Protection and Food Safety, Oldenburg, Germany (33.9-42502-04-12/0876). All procedures involving animals were carried out in accordance with the German Animal Welfare Act.

### RUSITEC Experiments

Four RUSITEC experiments were carried out using two RUSITEC systems with in total 12 fermenters. Inoculation of the fermentation vessels was carried out as described previously (Eger et al., 2018), with the modification that for each experiment rumen contents of one of the four cow were used. Briefly, rumen ingesta were squeezed through two layers of medical gauze (Gazin^®^, 80 cm × 5 cm, Lohmann & Rauscher International GmbH Co.KG, Rengsdorf, Germany) to separate the liquid and solid phase. Rumen contents were transported in preheated insulated containers to avoid heat losses. For each fermenter, a nylon bag (R712 Forage Bags *in situ*, ANKOM Technology, Gesellschaft für Analysetechnik HLS, Salzwedel, Germany) was filled with 70 g of solid rumen ingesta and placed in the inner fermentation vessel. A second nylon bag containing the daily feed was added. Feed bags contained 15 g of substrate. Components and chemical contents of the daily substrate are presented in Table 1. All components were shown to be free from glyphosate and aminomethylphosphonic acid (AMPA) before use (< 0.01 mg/kg, Wessling GmbH, Hannover). In each experiment, three fermentation vessels served as control, and one vessel each was assigned to one of the following nine treatments: 0.1 mg/l, 1.0 mg/l or 10 mg/l of pure glyphosate (40% N-(Phosphonomethyl) glycine, monoisopropylamine salt solution, Sigma-Aldrich, Darmstadt, Germany), 0.1 mg/l, 1.0 mg/l or 10 mg/l of glyphosate as Durano TF (052389-83/MOT, Bayer Crop Science, Langenfeld, Germany), or 0.1 mg/l, 1.0 mg/l or 10 mg/l of glyphosate as Roundup^®^ LB plus (024142-60/MOT, Nordland Agrar, Süderlügüm, Germany). The first two concentrations were chosen to meet the concentrations of 0.08 mg/d to 6.78 mg/d reported for *in vivo* uptake of glyphosate by von Soosten et al. (2016), the highest concentration exceeds *in vivo* uptake levels by 10-fold to provide a safety margin. The compounds were dissolved in methanol. The experiments consisted of an equilibration period of 6 days, a control day (day 7) and the experimental period from day 8 to day 16, during which, on each day either the respective glyphosate treatment was added to the fermentation vessels or the same amount of methanol (control vessels). A physiological buffer solution was infused into the fermenters to achieve a liquid turnover of once per day (Table 2). In order to measure microbial protein synthesis, ^15^N was included in the buffer solution (Table 2).

**Table 1:** Components and chemical composition of the daily substrate.

Components	[%] of the daily substrate
Dried grass silage	49.50
Dried maize silage	39.70
Cracked wheat	5.00
Cracked dried soya cake	5.00
Minerals (Vitamiral^®^ Grün)	0.80
Analysed chemical profile	[%] of dry matter
Organic matter	92.14
Crude protein	12.92
Crude fat	4.61
Crude fibre	25.86
Neutral detergent fibre	50.86
Acid detergent fibre	25.39
Acid detergent lignin	2.46

**Table 2:** Buffer composition.

Substance	[mmol/L]
NaCl	28
KCl	7.69
CaCl_2_ 2 H_2_O	0.216
MgCl_2_ 6 H_2_O	0.63
HCl (1 N)	0.5
NaH_2_PO_4_ H_2_O	10
Na_2_HPO_4_ 12 H_2_O	10
NH_4_Cl	5.0
NaHCO_3_	97.9
NH_4_Cl (98 At.% ^15^N)	0.07

### Measurements

The pH and redox potential were measured daily throughout the experiment (Digital-pH-Meter 646, Knick GmbH & Co. KG, Berlin, Germany, electrodes: InLab^®^ Routine, InLab^®^ Redox Pro, Mettler-Toledo GmbH, Gießen, Germany). At control day and during the experimental period, fluid flow-through of the fermenters was recorded daily and samples for the analysis of NH_3_-N and short-chain fatty acids (SCFA) were collected daily from the overflow. Short-chain fatty acid concentrations were measured by gas chromatography as described by Koch et al. (2006). The concentration of NH_3_-N was determined by photometry as described by Riede et al. (2013). From day 10 to day 13, feed bags were collected for the analysis of nutrient degradation. To determine the degradation of organic matter (OM), crude fat (XL), crude protein (XP), crude fibre (XF), neutral detergent fibre (NDF) and acid detergent fibre (ADF) feed residues from the 4 days were pooled, analysed by Weender analysis (Chair of Animal Nutrition, Technical University of Munich, Munich, Germany) and compared to undigested substrate.

### Microbial Protein Synthesis

From day 10 to day 16, 20 ml of fermenter fluid was collected daily for the separation of liquid-associated microorganism (LAM) and frozen at −20°C. Solid-associated microorganism (SAM) was collected from the feed bags at days 14 and 15 by methyl cellulose incubation (Boguhn et al., 2013) and also frozen until further treatment. Liquid samples and the methyl cellulose samples were then thawed and subjected to differential centrifugation (Brandt and Rohr, 1981). First samples were centrifuged at 2,000 × *g* for 5 min. The supernatant was again frozen at −20°C and the pellet was discarded. The supernatant was thawed in a fridge, centrifuged at 2,000 × *g* and 5 min again and the supernatant was collected in a new tube. The samples were then centrifuged at 27,500 × *g* for 15 min. The supernatants of LAM samples, which represented the fermenter liquid, were stored for later ^15^N-analysis, while the pellets representing LAMs or SAMs were washed with 0.9% NaCl three times. The remaining pellets were weighted and frozen at −20°C. For the analysis, LAM and SAM pellets and fermenter liquid were freeze-dried and grinded with a glass stick. The amount of ^15^N was analysed using a mass spectrometer (TracerMAT, Thermo Fisher Scientific GmbH, Dreieich Germany) after incineration in an elementary analyser (EA1108, Fisons Instruments GmbH, Mainz-Kastel, Germany).

N assimilation in LAMs was calculated using the following formula:


NLAM=N15input−15Noutput15Nplateau[mg/d]

Where ^15^N_input_ is input by the buffer solution; ^15^N_output_ is the content in the fermenter liquid; and ^15^N_plateau_ is the ^15^N percentage in the reference microbes.

The N assimilation in SAM was calculated using the following formula:


$$N_{SAM}=\frac{\left({}^{15}N_{FR}{-}^{15}N_{nat}\right).N_{FR}}{\left({}^{15}N_{SAM}{-}^{15}N_{nat}\right)}[\text{mg/d}]$$

Where ^15^N_FR_ is the measured ^15^N content in the feed residues; ^15^N_nat_ is the natural content of 0.3663%; N_FR_ is the N content of the feed residues; and ^15^N_SAM_ is the measured ^15^N content in the reference microbes.

Microbial protein synthesis was calculated by multiplying N content by 6.25 (Boguhn et al., 2006). The efficiency of microbial protein synthesis was calculated by dividing totally synthesised protein by fermented organic matter.

### Statistical Analysis of Fermentation Data

Statistical analysis was performed using the Statistical Analysis System for Windows SAS, version 9.4 by using the SAS Enterprise Guide version 7.1 (SAS Institute Inc., Cary, NC, United States). A three-factorial analysis of variance was applied by using the procedure mixed to determine effects of ‘treatment’ (=glyphosate formulation), ‘concentration’ and ‘day’ for parameters measured daily. Treatment and concentration were set as independent factors. Statistical analysis applies for days 7 to 16; however, only days 7 and 16 (before application and end of application) are displayed in graphs and tables to improve readability. For parameters obtained once per treatment or from pooled samples, only effects of ‘treatment’ and ‘concentration’ were analysed by two-factorial analysis of variance. The levels of significance were set at ^*^p < 0.05, ^**^p < 0.01 and ^***^p < 0.001. Data are presented as means ± SD.

### Microbiome DNA and Protein Extraction

For microbiome and metaproteome analysis, 10 ml of fermenter liquid was collected at days 10–16 and SAM was collected at days 14 and 15. Bacteria pellets were thawed and lysed, followed by protein and DNA extraction as previously described (Haange et al., 2020). Briefly, samples were resuspended in 1 ml lysis-buffer (50 mm Tris, 5 mm EDTA, 0.4% SDS, 50 mm NaCl and 1 mm PMSF, pH = 8). This was followed by lysis using a Fastprep 24 (MP Biomedicals, Santa Ana, CA, United States) set to three cycles, with each cycle consisting of 1 min disruption at a speed of 5.5 m/s and 1 min at rest. The resulting extract was heated in a thermomixer (Thermomixer comfort 5,355, Eppendorf, Hamburg, Germany) at 60°C with shaking at 1400 rpm for 15 min and spun at 10,000 × g at 4°C for 10 min. Supernatants, containing the DNA and protein content, were stored at −20°C.

For DNA purification, half of the supernatant was thawed and 260 μl 10 M ammonium acetate was added, mixed and incubated on ice for 5 min. Samples were then spun at 20,000 × g for 10 min at 4°C and the supernatants were kept. Isopropanol in equal volume was added to the supernatants, mixed thoroughly and incubated on ice for 30 min. The samples were then centrifuged at 20,000 × *g* for 15 min at 4°C to pellet the DNA. The pelleted DNA was washed with 10 μl pure ethanol, dried in a speed vac and finally dissolved overnight at 4°C in TE-Buffer (1 mm EDTA and 10 mm Tris, pH = 8). The dissolved DNA was purified and proteins removed using the QIAmp DNA Mini Kit (QIAGEN, United States) and following the manufacturer’s instructions. Finally, the DNA content recovered from each sample was quantified using NanoDrop (NanoDrop2000, Thermo Scientific, Rockford, IL, United States).

#### 16S rRNA Gene Sequencing

The 16S gene region V3 to V4 of Bacteria and Archaea was amplified with the universal primers 341F and 806R (5′-3′, forward: CCTACGGGAGGCAGCAG; reverse: GGACTACHVGGGTWTCTAAT; Takahashi et al., 2014) from the purified DNA samples. Amplicons were sequenced by an Illumina MiSeq DNA sequencer (Illumina, San Diego, United States). Amplification and sequencing were done by StarSeq GmbH (Mainz, Germany). Raw data analysis was done by StarSeq GmbH (Mainz, Germany) following their standard data analysis pipeline. Briefly, raw sequencing data were de-multiplexed, quality checked by FastQC and primers trimmed. Paired-end reads were joined, low-quality reads were removed, reads were corrected, chimaeras removed and Amplicon Sequence Variants (ASVs) were obtained by the deblur workflow. Taxonomy was annotated to the ASVs using the SILVA database (Quast et al., 2013). Read counts of ASVs were normalised and relative abundances determined, followed by taxonomic binning of ASVs using the R scripts Rhea (Lagkouvardos et al., 2017).

#### Metaproteomics

The concentration of protein in the supernatants from the protein and DNA extraction was determined with bicinchoninic acid assay according to the manufacturer’s instructions (Pierce™ BCA Protein Assay Kit, Thermo Fisher Scientific, Waltham, United States). Then, 100 μg protein was precipitated in acetone 1:5 (V/V) at −20°C overnight and then centrifuged (10 min, 14,000 × g). The precipitate was dissolved in Laemmli buffer and used for SDS-PAGE analysis, in-gel digestion and protein purification with ZipTip^®^ treatment (Haange et al., 2020). The peptide lysate concentration was determined by NanoDrop (NanoDrop2000, Thermo Fisher Scientific, Rockford, IL, United States) and 2 μg peptide lysate was injected into nanoHPLC (UltiMate 3,000 RSLCnano, Dionex, Thermo Fisher Scientific, Waltham, United States). Peptides were separated on a C18-reverse phase trapping column (C18 PepMap100, 300 μm × 5 mm, particle size 5 μm, nano viper, Thermo Fischer Scientific, Waltham, United States), followed by a C18-reverse phase analytical column (Acclaim PepMap^®^ 100, 75 μm × 25 cm, particle size 3 μm, nanoViper, Thermo Fisher Scientific, Waltham, United States; Haange et al., 2019). Mass spectrometric analysis of peptides was performed on a Q Exactive HF mass spectrometer (Thermo Fisher Scientific, Waltham, United States) coupled to a TriVersa NanoMate (Advion, Ltd., Harlow, United Kingdom) source in LC chip coupling mode. LC gradient, ionisation mode and mass spectrometry mode are described in Haange et al. (2019). Raw data were processed with Proteome Discoverer (v 2.2, Thermo Fischer Scientific, Waltham, United States). Parameters for Sequest HT search engine were set to: Trypsin (Full), Max. Missed Cleavage: 2, precursor mass tolerance: 10 ppm and fragment mass tolerance: 0.02 Da. Protein identification was done as previously described using a protein sequence data base constructed from all protein coding sequences of microbial genera identified in the 16S rRNA gene profiling as well as from plants commonly found in animal feed available on the UniProt (23.10.2017)^1^ data repository (Haange et al., 2019). Protein grouping was enabled, with protein group requiring at least one unique peptide. Relative abundance of protein groups was calculated as normalised intensity divided by the sum of all protein group intensities in the sample. Protein function and metabolic pathway relative abundances were calculated by summing all relative abundances of the associated protein groups.

### Quantification of Glyphosate in Reactor Medium

Quantification of glyphosate in the fermenter medium was done as previously described (Krause et al., 2020). Briefly, for the extraction of glyphosate, 1,000 μl of methanol:acetonitrile:water (2:3:1) was added to 100 μl of specimen. Samples were vortexed, sonicated and centrifuged at 14,000 × *g*. The supernatant was dried in a vacuum centrifuge (Concentrator Plus, Eppendorf AG Hamburg, Germany) and re-dissolved in 100 μl of Milli-Q water afterwards. A 10 μl of each sample of the resuspended extract was injected into a BEH Amide column (2.1 × 100 mm, 1.7 μm) supplied by Waters (Milford, United States). Chromatographic separation was performed with a gradient of solvent A (66% H_2_O, 33% acetonitrile, 10 mm ammonium acetate and 0.04% ammonium hydroxide; pH 9) and solvent B (10% H_2_O, 90% acetonitrile, 10 mm ammonium acetate and 0.04% ammonium hydroxide; pH 9). The LC was run at a constant flow rate of 0.4 ml/min. Initial equilibration for 2 min with 0% B, within 2.5 min gradient from 0 to 100% solvent B, hold 100% solvent B for 2 min, back to 0% solvent B for 3.4 min. Targeted multi-reaction monitoring (MRM) measurement of glyphosate was done on a QTRAP^®^ 5,500 (AB Sciex, Framingham, United States) in negative ionisation mode. Analyst^®^ Software (AB Sciex, Framingham, United States, version 1.6.2) was used for data acquisition and analysis.

### Statistical Analysis of Omics Data

Alpha-diversity and principal component analysis (PCA) were done in R (Ihaka and Gentleman, 1996) using the basic functions and the vegan package (Dixon, 2003). Statistics were done in R using in-house written scripts as previously described (Haange et al., 2020). Briefly, the statistical tests used were for complete sample data analysis Permutational Multivariate Analysis of Variance (PERMANOVA) using the adonis function in the vegan R package, and for single variables, Kruskal-Wallis group test followed by a post-hoc pairwise Dunn test. Where appropriate (number of tests >20), *p*-values were corrected for multi-testing by the Benjamini-Hochberg method (Benjamini and Hochberg, 1995). Heat maps were constructed with pheatmap R package (version 1.10)^2^ and all other figures were constructed using the R package ggplots2 (Wickham, 2011).

## Results

### Fermentation Parameters

**Figure 1:**
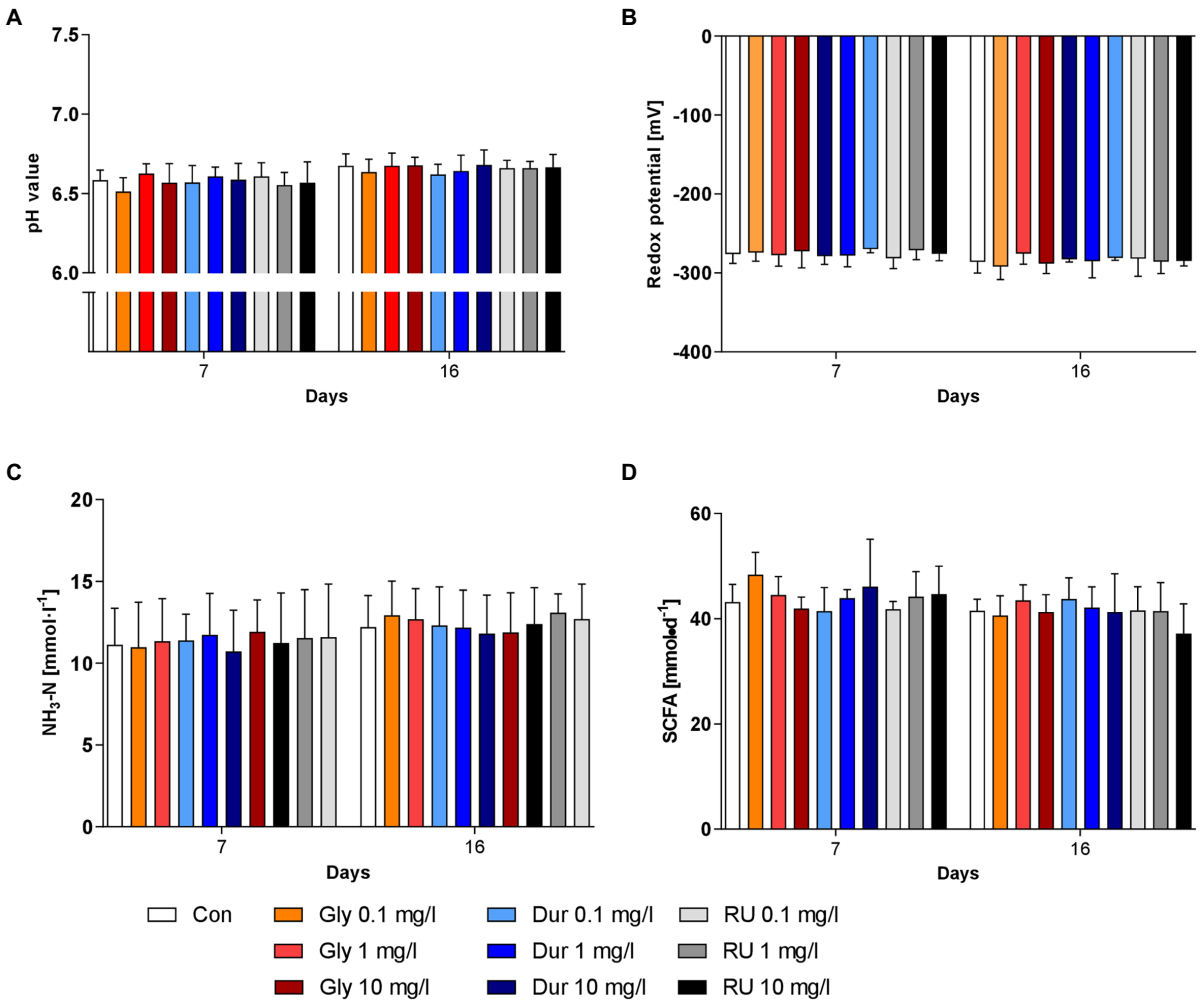
Effects of glyphosate on biochemical parameters. Effects of pure glyphosate (Gly), Durano TF (Dur) and Roundup^®^ LB plus (RU) at different concentrations on pH **(A)**, redox potential **(B)**, NH_3_-N concentration **(C)** and SCFA production **(D)** were compared to control group (Con) before addition (day 7) and after 9 days of addition at day 16. Data are presented as means ± SD.

The effects of different formulations and concentrations of glyphosate on rumen fermentation were assessed by measuring pH (Figure 1A), redox potential (Figure 1B), NH_3_-N concentration (Figure 1C) and production of SCFA (Figure 1D). None of these parameters were significantly affected by the factors ‘glyphosate formulation’ or ‘concentration’. Slight variations over time were observed for all four parameters. The pH value slightly increased over time (*p* < 0.001) with a value of 6.58 ± 0.09 (mean ± SD) at day 7 and 6.66 ± 0.07 at day 16 (Figure 1A). Although this difference was significant, the change in pH value was very small and all values were within the physiological range. The redox potential also varied significantly over time (*p* < 0.001); however, all values were within physiological levels (Figure 1B). The concentration range of NH_3_-N was between 11.4 ± 2.3 mmol/l at day 7 and 12.4 ± 1.9 mmol/l at day 16 (*p* < 0.001). The production of SCFA varied between 44.0 ± 4.5 mmol/d and 41.4 ± 4.3 mmol/d (effect of time *p* < 0.01). For all four mentioned parameters, no significant interactions were present between the factors. The molar proportions of the individual SCFAs were influenced neither by glyphosate formulation, nor by concentration, nor were interactions revealed. While the proportion of acetate remained unchanged at about 45–50% throughout the whole experiment, the proportions of propionate, butyrate, isobutyrate, valerate and isovalerate varied slightly over time (at least *p* < 0.05, means are presented in Supplementary Figure 1). The proportions of propionate ranged between 23.3 ± 3.1% and 23.6 ± 3.0%, butyrate accounted for 16.7 ± 1.1%–16.4 ± 1.5% of the SCFA mixture, while isobutyrate was below 1%. The proportion of valerate varied between 6.3 ± 0.9% and 6.6 ± 0.7% and the proportion of isovalerate between 4.5 ± 0.7% and 4.6 ± 0.8%. Although significant time-dependent alterations were observed for nearly all biochemical parameters, these were relatively small and most probable of low biological impact.

### Feed Degradation

The degradation of organic matter did not differ based on treatment, glyphosate concentration or interaction of both (Table 3). A degradation of 51.4–55.9% was observed during the 48 h incubation period. The degradation of crude fat was more variable with 26.0–39.5% (Table 3). The degradation of crude protein reached between 70.5 and 72.0% and between 38.5 and 44.4% of crude fibre were degraded (Table 3). Within the fibre fraction, 41.9–48.1% of NDF and 32.7–38.4% of ADF were degraded (Table 3). None of these parameters was significantly altered by treatment, glyphosate concentration or an interaction of both (Table 3).

**Table 3:** Nutrient degradation during the 48 h incubation period in the RUSITEC system.

Degradation of [%]	Treatment										
	Control	Glyphosate 0.1 mg/ml	Glyphosate 1.0 mg/ml	Glyphosate 10 mg/ml	Durano 0.1 mg/ml	Durano 1.0 mg/ml	Durano 10 mg/ml	Roundup 0.1 mg/ml	Roundup 1.0 mg/ml	Roundup 10 mg/ml	Pooled SD
Organic matter	53.1	52.8	53.9	53.8	51.4	52.4	52.4	52.9	55.9	53.7	3.3
Crude fat	33.0	26.0	39.5	32.3	28.0	35.5	33.1	29.2	32.1	37.2	9.5
Crude protein	71.6	70.9	72.7	70.5	71.8	70.6	70.7	71.7	72.7	71.3	5.0
Crude fibre	41.9	42.1	42.3	42.5	38.5	40.5	40.4	40.9	44.4	43.9	6.3
NDF^1^	44.9	45.4	44.9	45.8	41.9	43.3	43.5	44.6	48.1	45.3	7.3
ADF^2^	35.2	35.9	35.3	37.1	32.7	34.5	34.9	36.1	38.4	37.0	6.2

Degradation was determined from pooled samples from the experimental period. Two-factorial analysis of variance did not reveal significant differences.

1 Neutral detergent fibre.

2 Acid detergent fibre.

### Microbial Protein Synthesis

**Figure 2:**
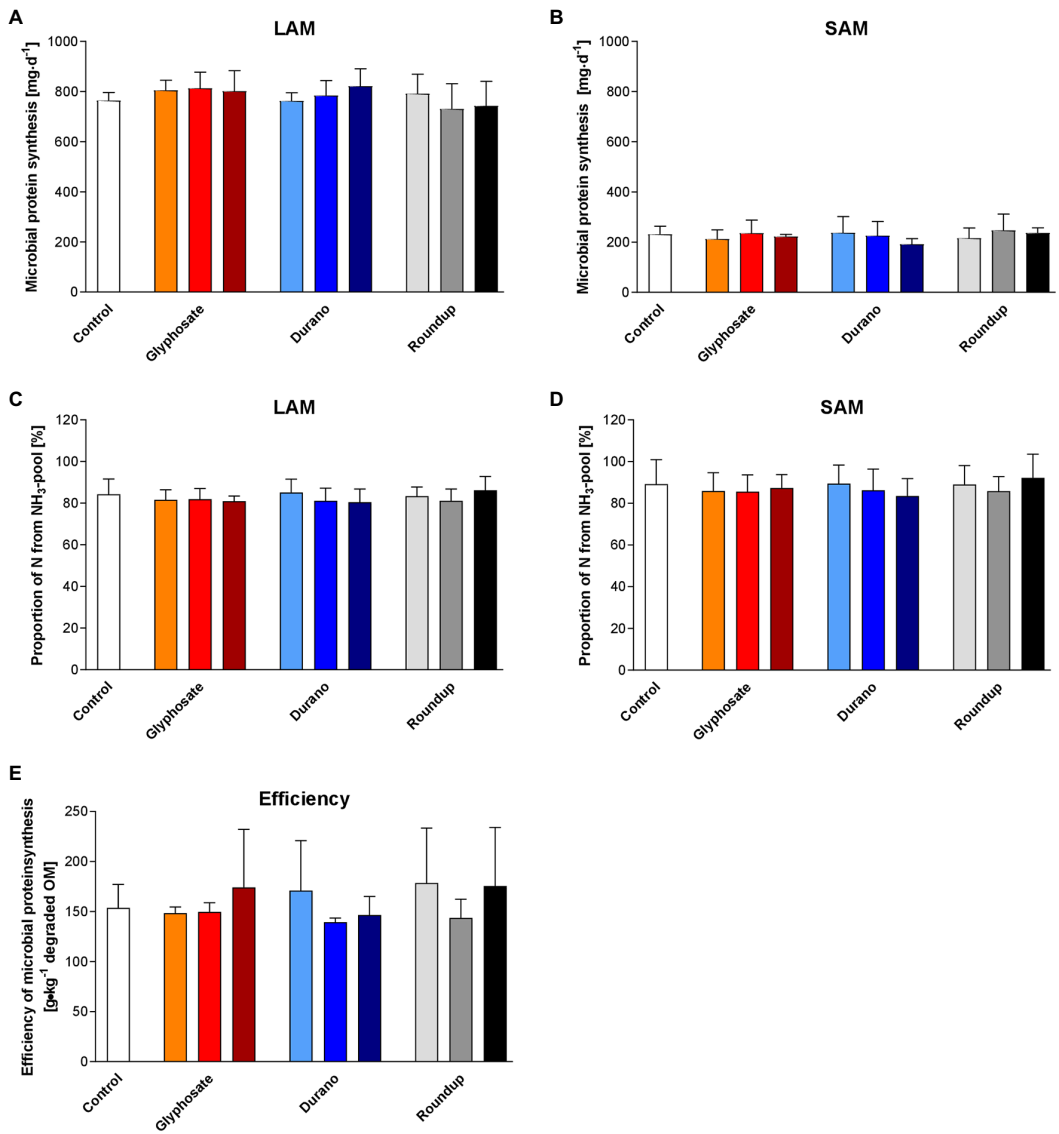
Effects of glyphosate on microbial protein synthesis. The effects of pure glyphosate, Durano TF and Roundup^®^ LB plus on microbial protein synthesis of liquid-associated microorganism (LAM; **A**), solid-associated microorganisms (SAM; **B**), the proportion of N derived from the NH_3_-N pool for LAM **(C)** and SAM **(D)** and the efficiency of microbial protein synthesis **(E)** were evaluated. For each formulation, three different concentrations were used as: 0.1 mg/l (orange, light blue and light grey), 1 mg/l (red, blue and grey) and 10 mg/l (dark red, dark blue and black). Data are presented as means ± SD.

Rumen microorganisms produced about 1 g of protein per day in each fermenter. Most of this protein (781.8 ± 66.5 mg/d) was produced by the LAM (Figure 2A), while SAM produced only about 225.0 ± 41.7 mg/d (Figure 2B). For both, LAM and SAM, more than 80% of N for microbial protein synthesis was derived from the NH_3_-pool (Figures 2C,D). The efficiency of microbial protein synthesis (Figure 2E) reached about 158.0 ± 35.5 g/kg degraded dry matter. None of the parameters for microbial protein synthesis was affected by treatment, glyphosate concentration or interaction of both.

### Microbial Community Structure

**Figure 3:**
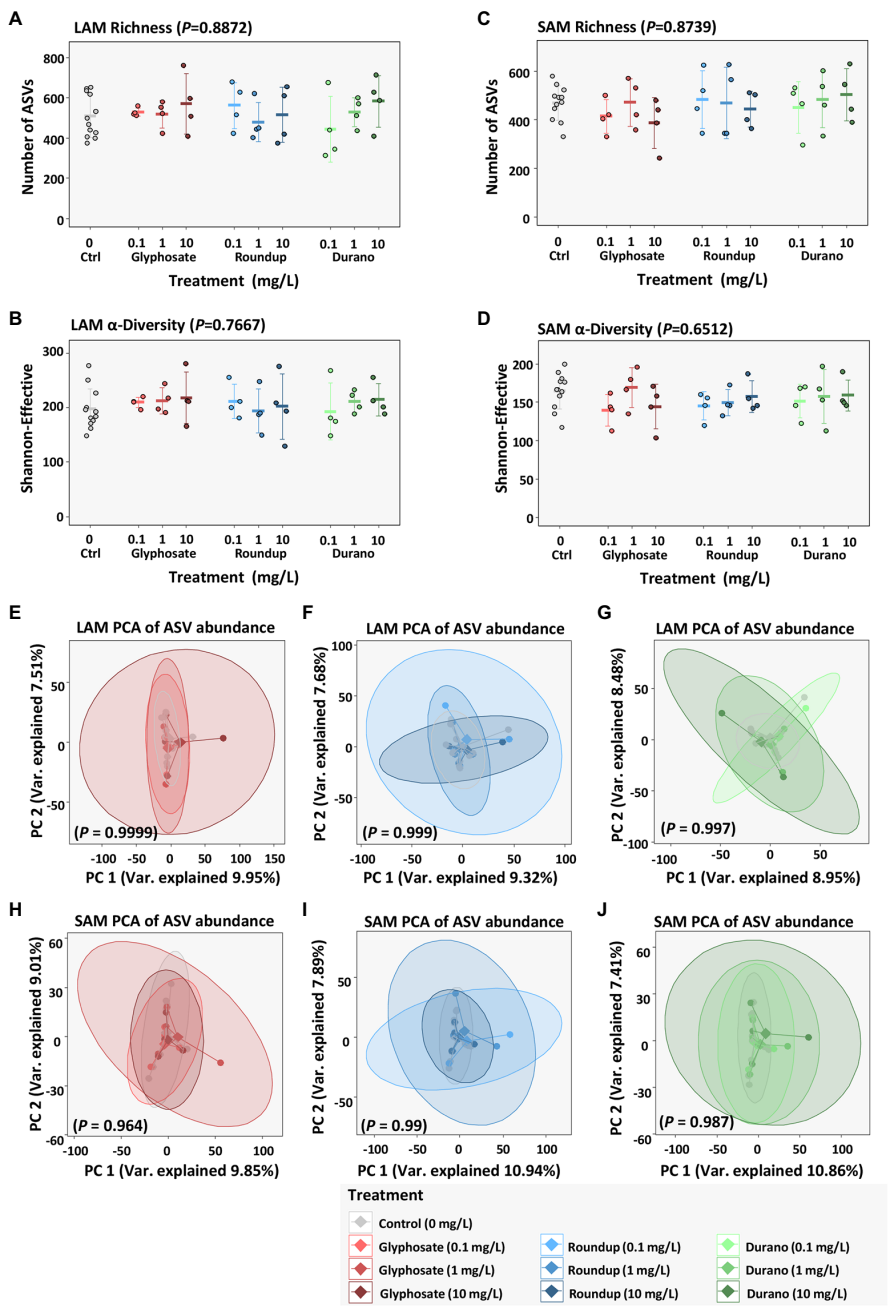
Influence of glyphosate and glyphosate formulations on diversity of bacterial communities based on abundances of amplicon sequencing variants (ASVs). LAM richness **(A)** and alpha-diversity **(B)** as well as SAM richness **(C)** and alpha-diversity **(D)**, beta-diversity based on principal component analysis (PCA) of LAM treated with pure glyphosate **(E)**, Roundup^®^ LB plus **(F)** and Durano TF **(G)** as well as SAM communities treated with pure glyphosate **(H)**, Roundup^®^ LB plus **(I)** and Durano TF **(J)**. For each formulation, three different concentrations were used as: 0.1 mg/l, 1 mg/l and 10 mg/l and compared to untreated controls.

To investigate any shifts in taxonomic structure of the microbiome associated with treatment with glyphosate or its formulations Durano and Roundup, 16S rRNA gene profiling was performed. The mean number of taxa based on amplicon sequencing variants (ASVs) was 508 ± 105 for LAM (Figure 3A) and 468 ± 70 for SAM (Figure 3B). There was no significant difference (LAM: *p* = 0.887, SAM: *p* = 0.874, Kruskal-Wallis test) in the number of taxa present for either the LAM or SAM when treated with glyphosate or a glyphosate formulation. A decrease in alpha-diversity, a combination of the number of taxa present in a sample and their distribution is often a sign of dysbiosis. Therefore, alpha-diversity based on Shannon-Effective was calculated (Figures 3C,D), but no significant change in Shannon-Effective between the control samples and those cultivated with different concentrations of glyphosate or a glyphosate formulation (LAM: *p* = 0.767, SAM: *p* = 0.651, Kruskal-Wallis test) was observed. To investigate if there was a global shift in taxonomic distribution between the control samples and the glyphosate and glyphosate formulation exposed microbial community’s, beta-diversity analysis based on principal component analysis was done. No clustering was observed between the control communities and those exposed to glyphosate at different concentrations of glyphosate. Neither for glyphosate, Durano nor Roundup exposure at the concentrations of 0.1 mg/l, 1 mg/l or 10 mg/l were significant differences in global community structure observed in the LAM (Figures 3E–G) or SAM (Figures 3H–J) communities.

**Figure 4:**
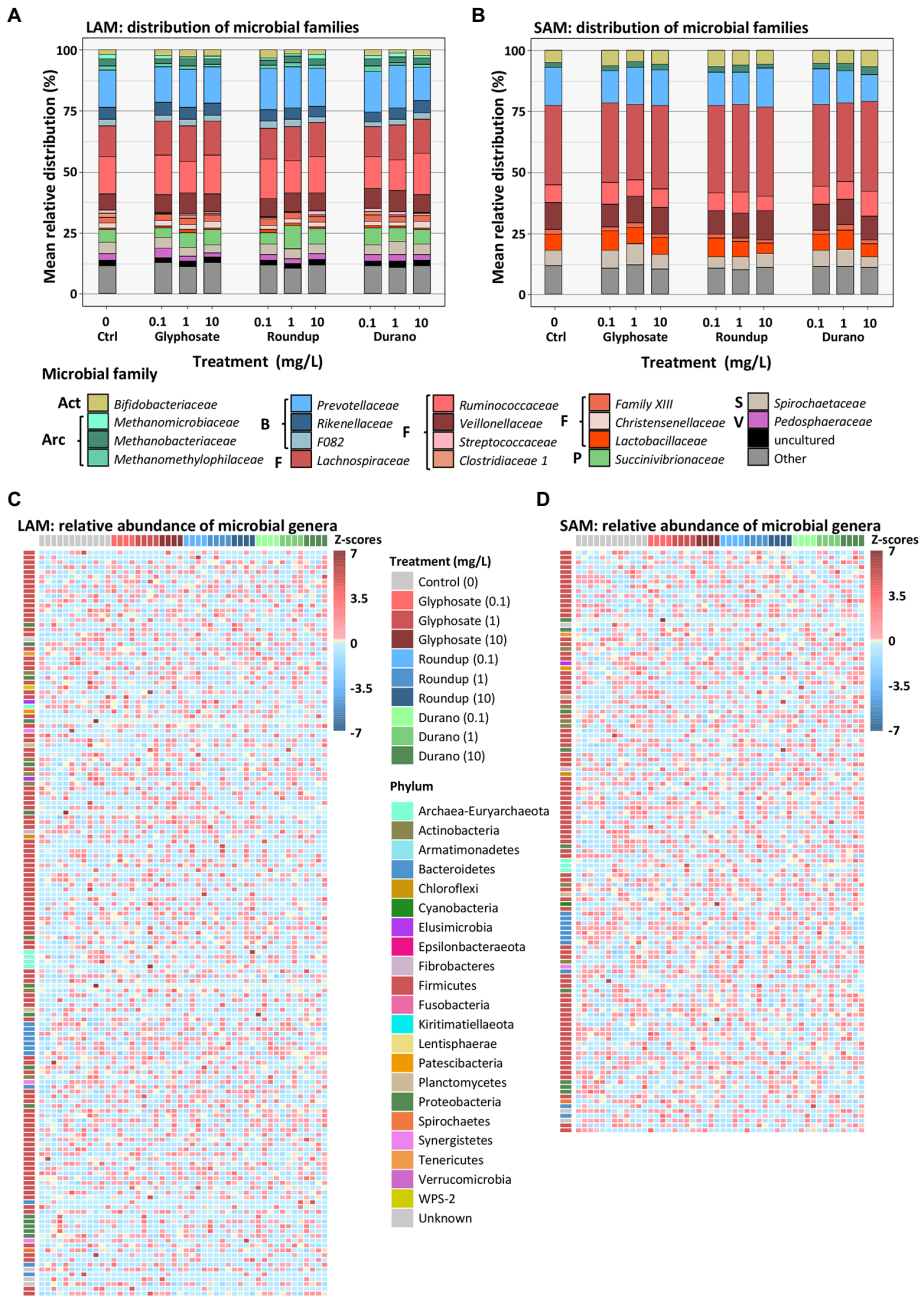
Relative distribution of microbial families in LAM **(A)** and SAM **(B)** communities as well as relative abundance of microbial genera in the LAM **(C)** and SAM **(D)** communities treated with pure glyphosate, Roundup^®^ LB plus and Durano TF. For each formulation, three different concentrations were used as: 0.1 mg/l, 1 mg/l and 10 mg/l and compared to untreated controls. **(A,B)**: Act = Actinobacteria, Arc = Archaea, B = Bacteroidetes, F = Firmicutes, S = Spirochaetota, V = Verrucomicrobia. The **(C,D)** depict relative abundance based on z-scores.

On the taxonomic level of family, the most abundant families in the non-treated LAM communities were from the Firmicutes *Ruminococcaceae* (15.1 ± 4.3%, mean ± SD), *Lachnospiraceae* (12.9 ± 3.6%) and *Veillonellaceae* (6.8 ± 2.1%), and from the Bacteroidetes the families *Prevotellaceae* (15.2 ± 3.6%), *Rikenellaceae* (4.9 ± 1.1%) and the F082 (2.6 ± 0.8%). Other abundant families included *Succinivibrionaceae* (5.4 ± 6.6%), *Spirochaetaceae* (4.7 ± 2.2%), *Bifidobacteriaceae* (2 ± 2.2%) and the archaea family *Methanobacteriaceae* (2.9 ± 1.6%; Figure 4A). The SAM non-treated community exhibited a different distribution in bacterial families than the LAM. Here, the most abundant families were *Lachnospiraceae* (32.6 ± 3.2%), *Prevotellaceae* (15.6 ± 2.8%) and *Veillonellaceae* (10.8 ± 1.2%), *Ruminococcaceae* (7.5 ± 2.5%), *Lactobacillaceae* (6.6 ± 2.7%), *Spirochaetaceae* (6.5 ± 1.6%), *Bifidobacteriaceae* (4.8 ± 1.8%) and the archaeal family *Methanobacteriaceae* (2 ± 0.6%; Figure 4B). No significant changes in relative abundance of any bacterial family were observed in either LAM or SAM communities with treatment with glyphosate, Durano or Roundup.

To investigate if changes in taxa distribution could only be observed at higher taxonomic resolution, an analysis on genus level was conducted. In total, we identified 155 genera in LAM (Figure 4C) and 121 genera in SAM (Figure 4D) with a relative abundance of at least 0.01% in one sample. No significant change in abundance was observed for any single genus in either LAM or SAM treated with glyphosate or a glyphosate formulation.

### Microbial Functional Analysis

**Figure 5:**
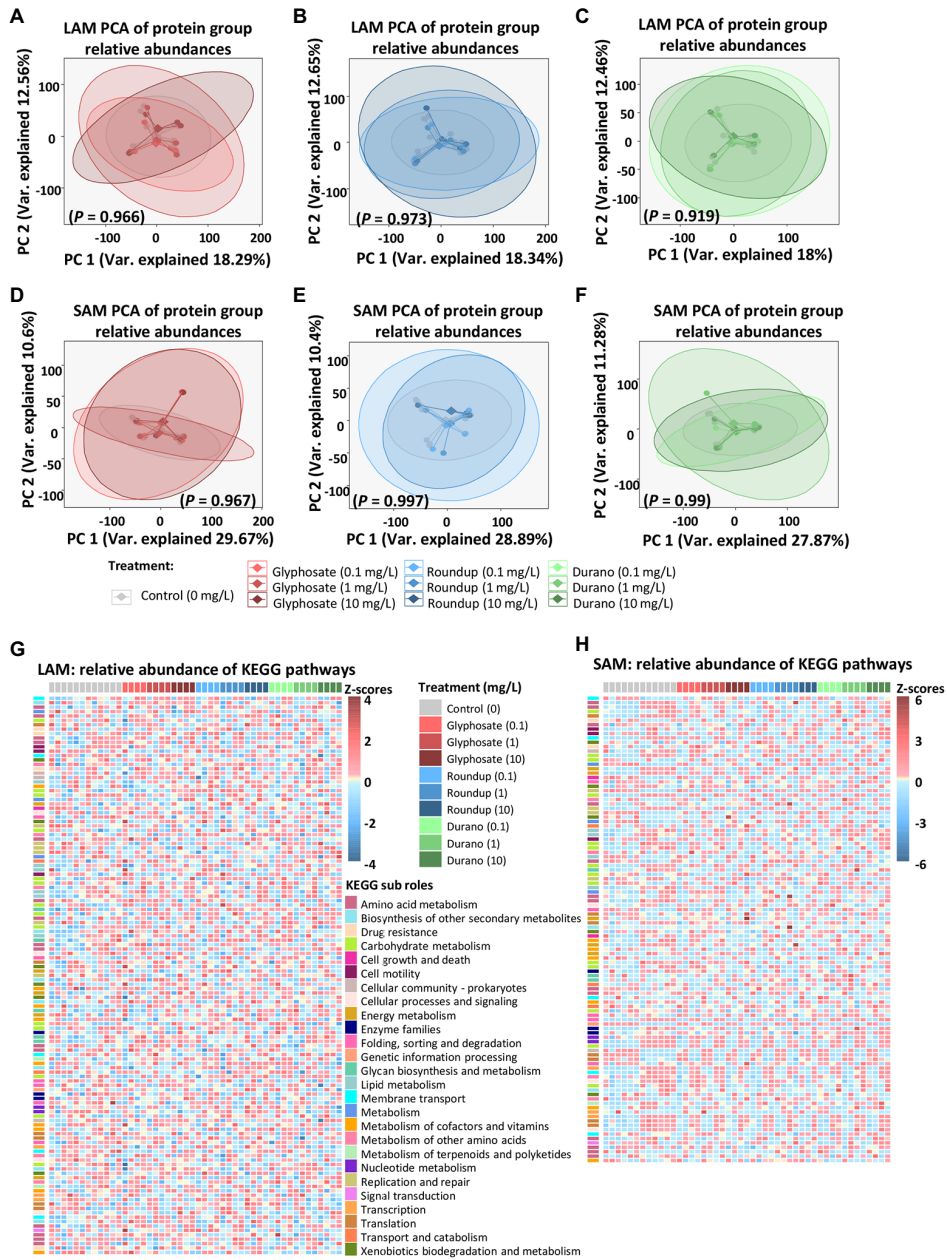
Influence of glyphosate and formulations on functions of microbial communities based on metaproteomic data. Principal component analysis (PCA) of protein group relative abundances of LAM treated with pure glyphosate **(A)**, Roundup^®^ LB plus **(B)** and Durano TF **(C)** as well as SAM communities treated with pure glyphosate **(D)**, Roundup^®^ LB plus **(E)** and Durano TF **(F)**. Relative abundances based on z-scores of KEGG metabolic pathways present in LAM **(G)** and SAM **(H)** communities. For each formulation, three different concentrations were used as: 0.1 mg/l, 1 mg/l and 10 mg/l and compared to untreated controls.

**Figure 6:**
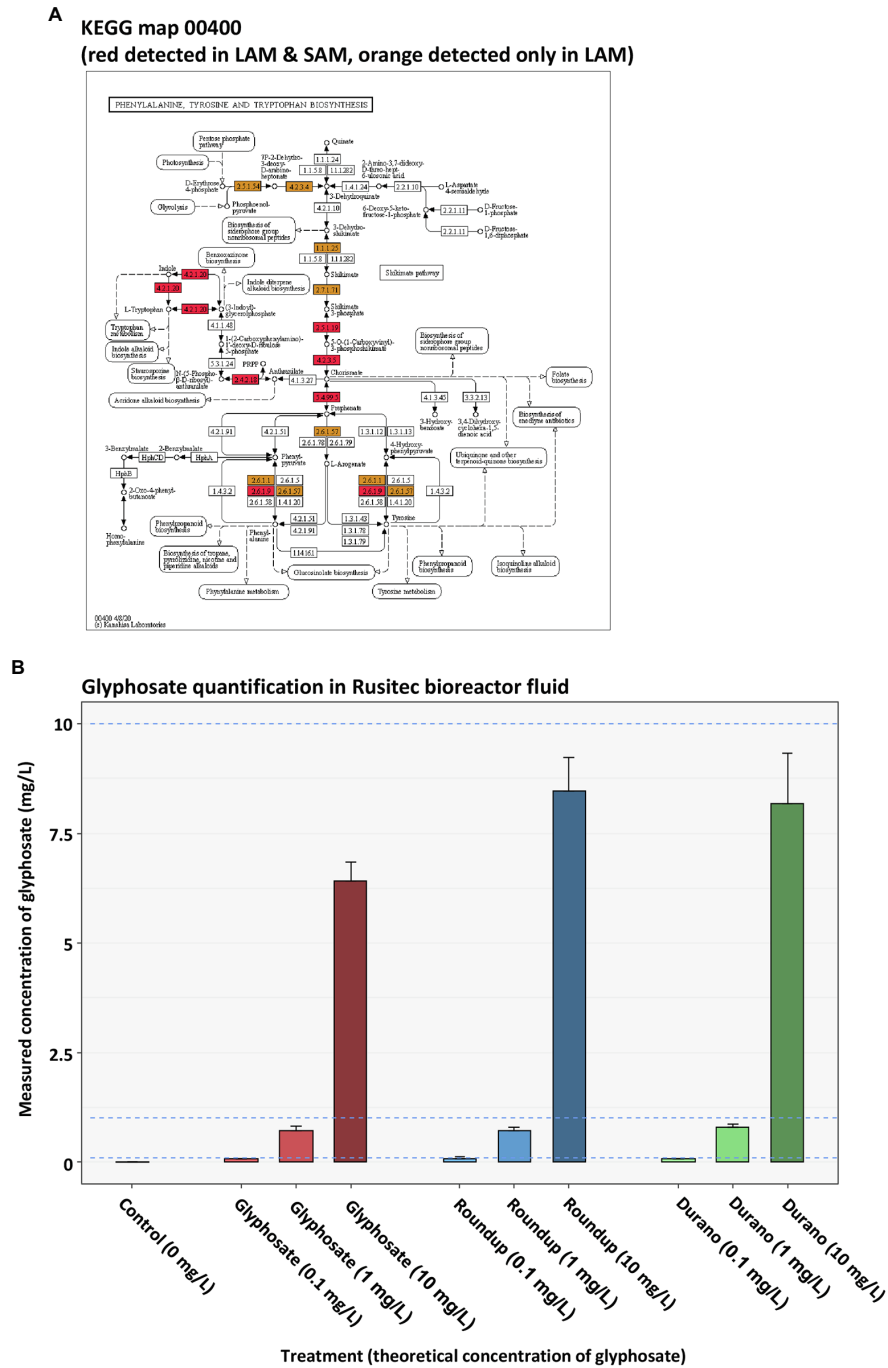
KEGG map 00400 of the phenylalanine, tyrosine and tryptophan biosynthesis **(A)** depicting all the protein functions identified in LAM and SAM (red) as well as only in LAM (orange). Quantification of glyphosate present in reactor medium at sampling time point **(B)**.

Metaproteomics was performed to analyse if glyphosate or glyphosate formulation exposure affected the functions exhibited by the LAM or SAM communities. In the LAM communities, a total of 8,052 microbial protein groups were detected, whereas in the SAM communities, 5,992 microbial protein groups were detected. To determine global metaproteome wide significant changes associated with glyphosate exposure or its formulations, principle component analysis was performed with the relative abundance of protein groups. PERMANOVA was used to determined significance. No global significant differences between the untreated control LAM (Figures 5A–C) or the untreated SAM communities (Figures 5D–F) with those treated with glyphosate, Durano or Roundup were observed. To investigate possible specific functional changes, protein groups were annotated to Kyoto Encyclopedia of Genes and Genomes (KEGG)^3^ (Ogata et al., 1999) metabolic pathways and the relative abundances summed and compared between the treatment groups. In total, 134 KEGG metabolic pathways with a functional coverage of at least 10% were identified in LAM (Figure 5G), while 106 were seen in SAM (Figure 5H). Glyphosate, Durano or Roundup treatment at any of the three concentrations (0.1 mg/l, 1 mg/l and 10 mg/l) did not significantly enrich any KEGG functional pathway when compared to the untreated controls. The KEGG pathway map 00400 was investigated more closely since it included the shikimate pathways and the 5-enolpyruvylshikimate-3-phosphate (EPSP) synthase, which is the specific target for glyphosate. We identified 16 KEGG protein functions including the EPSP synthase in the metaproteomes of LAM and SAM (Figure 6A), but none of them were significantly altered depending on the abundance by glyphosate, Durano or Roundup treatment.

### Glyphosate Quantification and Correlation Analysis

One potential reason for the lack of changes in the taxonomic or functional community structure of LAM or SAM observed after glyphosate, or glyphosate formulation treatment, could be a potential degradation of glyphosate in the microbial community. To control for this, we quantified glyphosate in the bioreactor supernatant by targeted multi-reaction monitoring mass spectrometry. As expected, in the untreated control samples, no glyphosate was detected (Figure 6B). The spiked in concentration in the bioreactor medium was 10 mg/l, 1 mg/l and 0.1 mg/l. In the glyphosate or glyphosate formulation treated bioreactors, we detected somewhat lower levels of glyphosate than expected. The concentrations in the bioreactors with pure glyphosate were 6.414 mg/l ± 0.438 mg/l, 0.717 mg/l ± 0.105 mg/l and 0.068 mg/l ± 0.009 mg/l. In the bioreactors with Roundup, the measured concentrations were 8.478 mg/l ± 0.753 mg/l, 0.791 mg/l ± 0.088 mg/l and 0.074 mg/l ± 0.011 mg/l. In the bioreactors containing Durano, glyphosate concentrations after the run were measured at 8.179 mg/l ± 1.152 mg/l, 0.791 mg/l ± 0.088 mg/l and 0.074 mg/l ± 0.011 mg/l. These determined concentrations values were consistent with no or little degradation of the glyphosate when taking the accuracy tolerance (78% relative recovery) for the glyphosate quantification method into account. This suggested that the majority of glyphosate was still present in the medium. To reveal potential correlations among glyphosate concentration in the fermenter, microbial abundances and functions, the measured glyphosate values were correlated with the relative abundance of the microbial genera detected by 16S rRNA gene profiling and the relative abundance of KEGG metabolic pathways in the metaproteomics study. No significant positive or negative correlations between the concentration of glyphosate and the abundance of genera or KEGG pathways in the LAM or SAM were identified (data not shown). In addition, the relative abundances of protein functions of the KEGG 00400 metabolic pathway map (aromatic amino acid biosynthesis) identified in LAM and SAM were not significantly positively or negatively associated with the glyphosate concentration (data not shown).

## Discussion

The rumen microbiota consists of bacteria, fungi, protozoa and archaea, containing members, which are potentially vulnerable to inhibition of the 5-enolpyruvylshikimate-3-phosphate pathway by glyphosate and/or adverse effects of adjuvants in commercial formulations. Therefore, the aim of this study was to investigate the effects of pure glyphosate and two commercially available formulations on rumen fermentation and rumen microbiota.

Neither the type of glyphosate applied, nor the concentration of the three formulations affected biochemical parameters such as pH, redox potential and concentration of NH_3_-N or SCFA production. Previous studies on the impact of glyphosate on these parameters are variable. The mentioned parameters were not altered by application of Roundup Ultra^®^ (0.77 mg/d) in sheep (Hüther et al., 2005). Administration of Roundup^®^ LB plus to a bioreactor model for the pig colon microbiota did also not change SCFA production (Krause et al., 2020). In contrast, Riede et al. (2016) applied Plantaclean^®^ 360 in two doses (about 0.46 mg/l and 3.9 mg/l) in a RUSITEC-trial and detected no effects on pH, redox potential and most SCFAs, but reported a decrease in NH_3_-N concentration and in propionate proportion, a tendency for increased total SCFA and acetate production, and an increase in isovalerate proportion. *In vivo*, Billenkamp et al. (2021) observed no effects of Roundup Record^®^ [about 80 mg/d (Schnabel et al., 2017)] on pH, NH_3_-N and the proportions of the main SCFA; however, total SCFA production and the proportion of isovalerate decreased. Summarising these studies, there were no consistent effects of different glyphosate formulations on rumen fermentation parameters. Glyphosate dosages in these studies were close to our medium dosage. Although no previous study comparing several formulations is available, the results of the mentioned studies are based on different formulations and agree with our result that there is no difference among glyphosate formulations.

Small variations in biochemical parameters were observed over time, e.g., pH varied about 0.08 units during the experimental period. Comparable variations have been observed in other Rusitec experiments without glyphosate additions (Orton et al., 2020) and are far below diurnal variations observed *in vivo* (Leedle et al., 1982). Therefore, these observations are probably of negligible biological relevance.

Microbial protein synthesis was to our knowledge not measured previously under glyphosate treatment. However, the lack of effect by glyphosate on microbial protein synthesis agrees with the stable NH_3_-N concentration. The degradation of organic matter, crude nutrients and fibre fractions was also unaffected by glyphosate in the present study. This agrees with the results on *in sacco* degradation of dry matter and NDF in sheep (Hüther et al., 2005).

Previous studies indicated an effect of glyphosate on bacterial communities, e.g., in soil (Lancaster et al., 2010), in the honeybee gut (Motta et al., 2018; Blot et al., 2019) or in the rat cecum (Mesnage et al., 2021). Therefore, we also investigated the rumen bacterial community, its metabolic activity and the potential inhibition of the EPSP pathway. We did not observe any effect of glyphosate, Durano or Roundup on the abundance of bacterial families and genera, nor on alpha or beta-diversity of the prokaryotic community.

Previous reports on the effects of glyphosate on rumen microorganisms were mainly based on short-time studies using isolated cultures of single strains or batch cultures (Krüger et al., 2013; Ackermann et al., 2015). The RUSITEC system, which was used here, is a more complex system, which is able to maintain the core bacterial community of the rumen over a longer time (Wetzels et al., 2018). Our results agree with previous RUSITEC studies, which observed only minor effects of glyphosate on the rumen bacterial population and no effects on the growth of pathogenic bacteria (Riede et al., 2016; Bote et al., 2019). In a recent *in vivo* study with dairy cattle, Billenkamp et al. (2021) observed differences for only four taxa, which, however, appeared to be linked closer to experimental conditions than to glyphosate application. One of the formulations used in the present study, Roundup^®^ LB plus was also tested in a pig colon microbiota *in vitro* model, where also no effect on the microbial community composition was detected (Krause et al., 2020). Metaproteome analysis confirmed the presence of the enzymes of the shikimate pathway and the EPSP synthase in the rumen microbiota; however, expression of these enzymes was not influenced by addition of pure glyphosate or its formulations at any concentration, which also agrees with the study by Krause et al. (2020) for the pig gut. Until now, there is no study available using untargeted metabolomics to focus on the end products of microbial fermentation pathways, this might be a future approach to support the current observations. Comparing the results of the mentioned studies, it appears that the effects of glyphosate on microbiota may be more pronounced, when single strains or simple microbial communities are investigated, while for more diverse communities, such as in the RUSITEC system or in the rumen *in vivo*, glyphosate effects are rarely observed. In addition, microbiomes in amino acid poor environments, such as soil or the honeybee gut, may rely more heavily on synthesis of aromatic amino acids, and therefore could be more susceptible to glyphosate inhibition. In contrast, microbiomes in amino acid rich environments such as the stomach and gut of livestock, especially if the host livestock is provided with protein-rich feed, may be resilient towards glyphosate inhibition.

In conclusion, glyphosate treatment, independent whether pure glyphosate, or the formulation Durano TF or Roundup^®^ LB plus was applied, did neither influence the rumen procaryotic community, nor its fermentation, nor expression of the EPSP synthase pathway in the RUSITEC system even in concentrations far above common intake levels. This underlines the high stability of the complex rumen microbial community.

## Data Availability Statement

The datasets presented in this study can be found in online repositories. The names of the repository/repositories and accession number(s) can be found at: ProteomeXchange Consortium *via* the PRIDE partner repository with the dataset identifier PXD032166. 16S rRNA gene sequencing data was deposited in the SRA repository and can be found under the identifier PRJNA802980.

## Ethics Statement

The animal study was reviewed and approved by Lower Saxony State Office for Costumer Protection and Food Safety (approval number: 33.9-42502-04-12/0876).

## Author Contributions

GB and MvB planned the study and acquired the funding. GB and SR planned the experiments. DS and SR were involved in performance of the Rusitec experiments. S-BH did 16S rRNA gene analysis. S-BH and NJ performed proteomics analysis. BE and UR-K did glyphosate measurements. SR, KR, MB and S-BH analysed the data. MB and S-BH wrote the paper. All authors approved the final version of the manuscript.

## Funding

This project was funded by the BMEL (BLE grant numbers 314-06.01-2815HS016 and 314-06.01-2815HS018). MvB is grateful for the support by Novo Nordisk Foundation grant NNF21OC0066551.

## Conflict of Interest

The authors declare that the research was conducted in the absence of any commercial or financial relationships that could be construed as a potential conflict of interest.

## Publisher’s Note

All claims expressed in this article are solely those of the authors and do not necessarily represent those of their affiliated organizations, or those of the publisher, the editors and the reviewers. Any product that may be evaluated in this article, or claim that may be made by its manufacturer, is not guaranteed or endorsed by the publisher.

## Footnotes

^1^ http://www.uniprot.org

^2^ http://cran.r-project.org/web/packages/pheatmap/index.html

^3^ https://www.genome.jp/kegg/
