# Supplementary figures and images for: Effects of Different Formulations of Glyphosate on Rumen Microbial Metabolism and Bacterial Community Composition in the Rumen Simulation Technique System
Source: Front Microbiol. 2022 Apr 29;13:873101. doi: 10.3389/fmicb.2022.873101 (PMC9100596; doi:10.3389/fmicb.2022.873101)

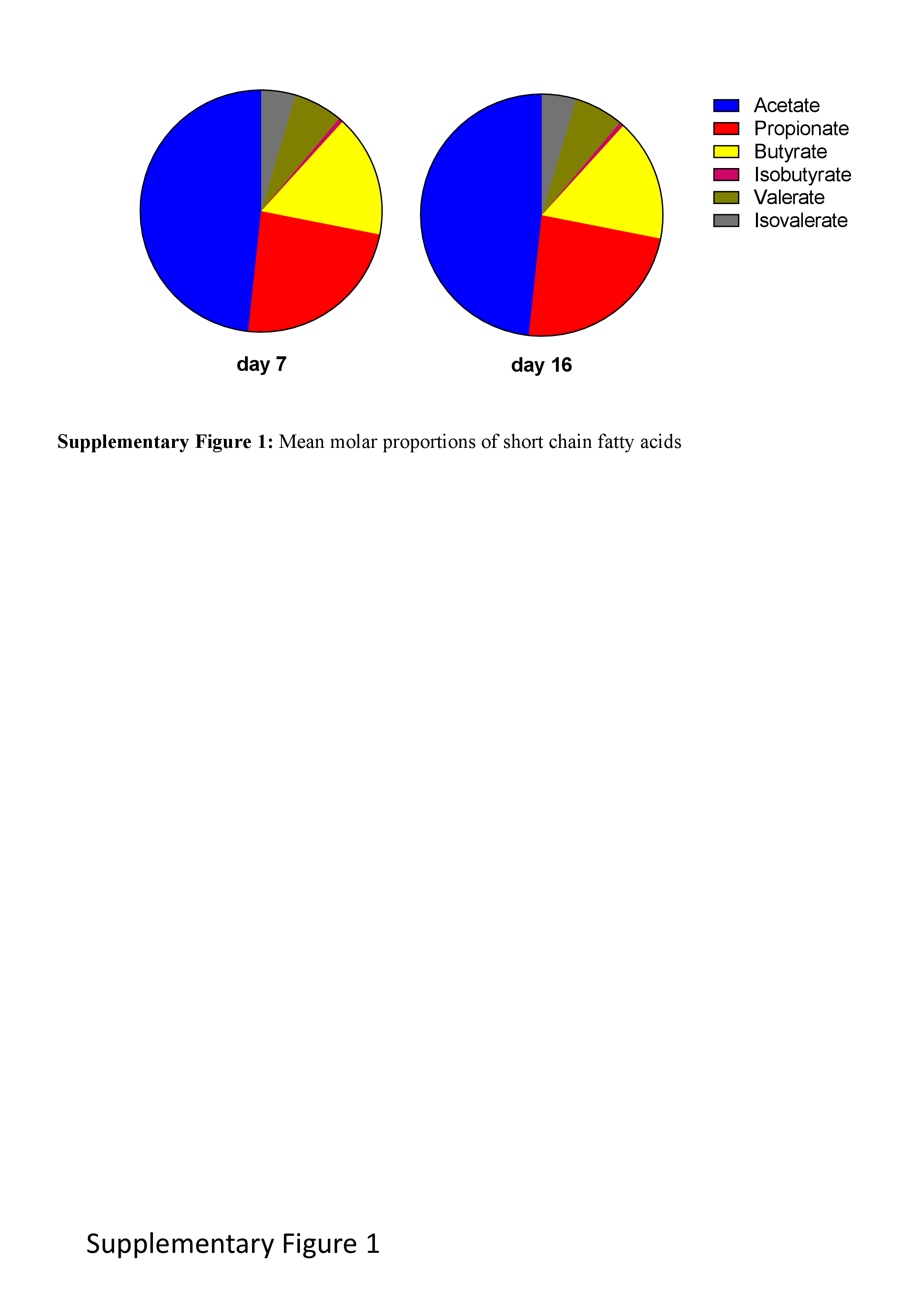

Supplement: Supplementary file 1 [file Image_1.TIF]
